# FMCW LiDAR System to Reduce Hardware Complexity and Post-Processing Techniques to Improve Distance Resolution

**DOI:** 10.3390/s20226676

**Published:** 2020-11-22

**Authors:** Chankyu Kim, Yunho Jung, Seongjoo Lee

**Affiliations:** 1 The Department of Information and Communication Engineering, Sejong University, Gunja-dong, Gwangjin-gu, Seoul 05006, Korea; peperman71@itsoc.sejong.ac.kr; 2 School of Electronics and Information Engineering, Korea Aerospace University, Goyang-si 10540, Korea; yjung@kau.ac.kr

**Keywords:** FMCW, LiDAR, FFT, hardware complexity, FPGA, FMCW LiDAR, Digital Down Convert

## Abstract

As the autonomous driving technology develops, research on related sensors is also being actively conducted. One system that is widely used today uses a light source with a wavelength in the 905 nm band for the pulse Light Detection And Ranging (LiDAR) system. This has the disadvantages of being harmful to the human eye and in making digital signal processing difficult at high sampling rates. The Frequency Modulated Continuous Wave (FMCW) LiDAR system has been proposed as an alternative. However, the FMCW LiDAR is formed with a high beat frequency by a method different from that of the FMCW Radar, which causes a hardware burden on the FFT (Fast Fourier Transform) module for interpreting the beat frequency information. In this paper, the FFT module that may occur in the FMCW LiDAR using Digital Down Convert (DDC) technology is extracted at 256 points, which is $2^{5}$ times smaller than the existing 8192 points, and the beat frequency is 0 to 50 m at 3 cm intervals. As a result of generating and restoring the distance, the performance of 0.03 m Root Mean Square Error (RMSE) compared to the conventional one was confirmed. In this process, the hardware module was directly mounted and verified on the FPGA. In the case of the Simple Threshold-Constant False Alarm Rate (ST-CFAR) provided, the RMSE was measured by generating beat frequencies from 0 to 50 m at 1 cm intervals, and as a result, the result of 0.019 m was confirmed at 0.03 m in the past.

## 1. Introduction

As various autonomous vehicles, such as autonomous vehicles and unmanned autonomous vehicles, are rapidly developed and utilized in various industrial fields, research is being actively conducted [1,2,3]. As a result, various studies have been conducted on various sensors, which are indispensable and should be applied to autonomous vehicles [4,5].

Various sensors, such as LiDAR, Radars, and ultrasonic sensors are applied to autonomous vehicles and for reliable detection of the surrounding conditions of autonomous vehicles according to various weather conditions and illuminance. There is a tendency for technologies that combine these to be used. Among these sensors, the LiDAR sensor is a sensor that detects an object and maps the distance and is used in various fields such as autonomous vehicles, three-dimensional (3D) aerial maps, factory systems, and atmospheric analysis [6,7,8,9].

Among the various LiDAR systems, a typical system used for autonomous vehicles is the pulse LiDAR system. The pulse rider emits a short pulse laser, measures the time interval and angle of the received reflected light signal and measures the distance of the object. [10] However, many such pulse LiDAR use light sources with wavelengths in the 905 nm band [11]. The 905 nm pulse LiDAR has problems such as interference between the reflected light of the sun and other light sources and has a fatal drawback that is harmful to the human retina [12]. Also, assuming that there is an object at a distance of 3 cm, using a pulse rider system with a distance resolution of 3 cm as an example, the time for the optical signal to be reflected back by the object is defined by Equation (1):

(1) $$T=\frac{d\cdot 2}{C}=\frac{3\;\mathrm{cm}\cdot 2}{3\cdot 10^{10}\;\mathrm{cm}/s}=0.2ns$$

In this case, it has a speed of at least 5 GHz and very precise sampling is performed. Having a high sampling rate makes digital signal processing difficult and results in very high hardware implementation costs.

An alternative FMCW LiDAR system has been proposed using a wavelength in the 1550 nm band, which is safe for the human eye, and has the advantage of lower peak power when compared to pulse technology [13,14,15,16]. Since then, various studies on the FMCW LiDAR system have been conducted. The FMCW method is already widely used in radar, but LiDAR has another point in that it uses the Chirp frequency modulation signal as an optical signal. It is a system that operates on a same principle in that it measures the distance of an object, while measuring the phase and frequency of the Chirp that returned after that.

The FFT module is used here to perform a frequency domain transform to extract the distance information from the FMCW system. However, in the case of the FMCW LiDAR system, the beat frequency generated by the transmission/reception chirp is formed high by a method different from the radar, which increases the load on the FFT module.

Section 2 of this paper describes the traditional FMCW LiDAR system and describes the principles of FMCW LiDAR. Section 3 provides a system that uses DDC technology and a lower spec FFT module to reduce the hardware load that can be displayed on the FMCW LiDAR system. Section 4 introduces ST-CFAR (Simple Threshold-Constant False Alarm Rate), which provides distance information that is better than the distance resolution determined by the system’s Chirping Bandwidth. Section 5 describes the process of implementing the proposed system in hardware. Section 6 applies the system and algorithm proposed in this paper to restore the beat frequency through experiments, compare it with the existing beat frequency, and compare the error rate when converted to distance information. Finally, Section 7 concludes the paper.

## 2. System Overview

This section describes the traditional FMCW LiDAR system. In addition, the principle of FMCW LiDAR technology will be explained. The FMCW method is a technology that has been used for quite some time in Radar. FMCW detects the distance, velocity, and angle to the target by receiving the signal that the transmitted waveform is reflected by the target and processing the signal. The FMCW LiDAR system also judges an object based on this principle and has a waveform whose frequency changes linearly with time. When the transmitted signal is reflected by the target and is received, the time-delayed optical signal is received in proportion to the distance to the target. At this time, the frequency difference between the transmission signal and the reception signal is defined as the Beat Frequency, and this Beat Frequency is analyzed to grasp the distance to the target. The structure of a traditional FMCW LiDAR system can be broadly classified into Direct Detection Architecture, Coherent Heterodyne Detection Architecture, and Homodyne Self-Chirped Detection Architecture.

**Figure 1:**
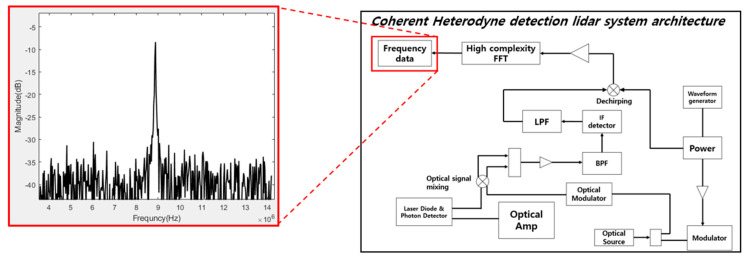
Traditional Coherent Heterodyne Detection FMCW LiDAR Architecture.

In this paper, we provide a modified structure targeting the Coherent Heterodyne Detection Architecture with the structure shown in Figure 1.

The left side of Figure 1 shows the result of restoring the frequency spectrum of the beat frequency signal with the FMCW LiDAR system, based on research conducted in the past [17]. In the case of the traditional FMCW LiDAR system, all three structures have a high Beat Frequency, which puts a lot of strain on the FFT of the DSP (Digital Signal Process) block to analyze this on the frequency domain. In order to improve this, we propose a method in this paper to reduce the load on the FFT module by applying DDC to the Beat Frequency collected via ADC.

## 3. Proposed System

This section provides a system that uses DDC technology and a lower spec FFT module to reduce the hardware burden that can be displayed on the FMCW LiDAR system. In this paper, we will cite various FMCW LiDAR systems that have been studied in the past, and proceed with the explanation of the system on the assumption that the reflected signal inside the system is received and the beat frequency is generated [18,19].

Equation (2) is an equation for converting the distance from the FMCW system, where *T_b_* is the sweep time, *C* is the constant of light, Δ*F* is the chirping bandwidth, and fb is the beat frequency. The value that changes with the distance from the FMCW LiDAR system according to Equation (2) is the beat frequency, which is the main lobe on the frequency spectrum. Due to the characteristics of FMCW LiDAR, which uses optical signals, it is possible to accurately distinguish between side lobes and main lobes even in heavily noisy situations, but these characteristics can be used to perform DDC. If only the FFT point of the main lobe at other times is restored and output, the distance information can be extracted.

(2) $$R=\frac{f_{b}\cdot T_{b}\cdot C}{2\Delta f}$$

**Figure 2:**
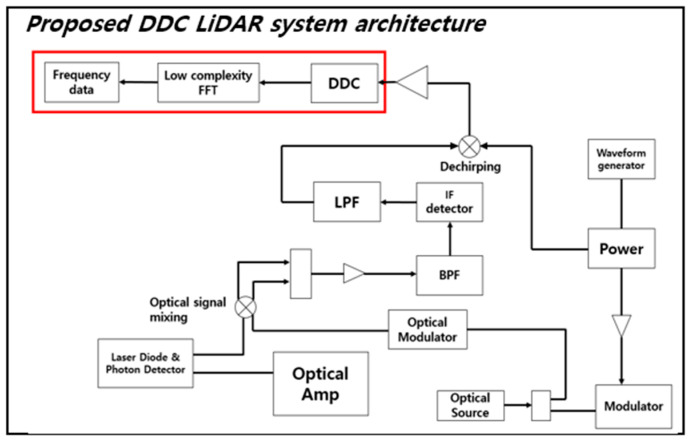
Proposed Coherent Heterodyne Detection FMCW LiDAR Architecture.

**Figure 3:**
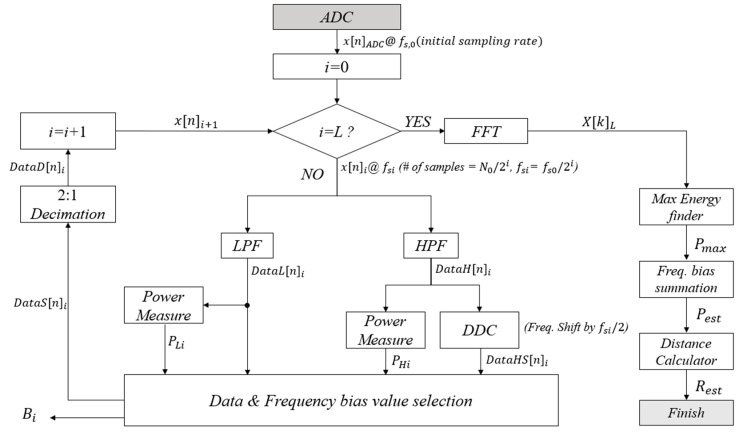
Proposed DDC FMCW LiDAR System’s Flow Chart.

In the case of the conventional FMCW LiDAR system, as shown in Figure 1, the Beat Frequency can be seen entering the input of the FFT block immediately after passing through the ADC, and then the result is used to perform Data Analysis. Since the center frequency of the Beat Frequency is formed very high in the process, the High complexity FFT module is used. On the other hand, Figure 2 shows the structure of FMCW LiDAR newly proposed in this paper. Other structures can be seen to match the traditional FMCW LiDAR system, but in the proposed system the DDC module is adopted in order to reduce the number of points of FFT module by finding the information frequency band of ADC data. Since the center frequency is shifted in the low frequency band via the DDC, a low complexity FFT block can be used. The process of the DDC block has the structure shown in Figure 3. In order to execute DDC, the beat frequency signals that have passed through the ADC are passed through the digital filters LPF and HPF, respectively, and the power is compared. At this time, the cut-off region of the LPF and HPF is set to half the sampling rate of the ADC to design the filter. Since the process of obtaining the signal power simply divides the cumulative total of the signals, when measuring the power of the signal that has passed through each filter, the one with the larger power belongs to the main lobe due to the characteristics of the FMCW LiDAR signal. From Figure 3 the iteration number of loop, *L*, is a parameter value determined by how the user wants to reduce the hardware complexity of the FFT module.

(3) $$DataL{\left[n\right]}_{i}=\;\overset{8}{\underset{k=0}{\sum }}Coeff_{L}\left[k\right]\cdot x{\left[n-k\right]}_{i}$$

(4) $$\;\;DataH{\left[n\right]}_{i}=\;\overset{8}{\underset{k=0}{\sum }}Coeff_{H}\left[k\right]\cdot x{\left[n-k\right]}_{i}$$

(5) $$\;\;\;\;\;\;\;\;\;\;\;\;P_{Li}=\;\;\overset{N_{i}-1}{\underset{k=0}{\sum }}{\left(DataL\left[k\right]\right)}^{2}\;\;\;\;\;\;\;\;\;\;\left(N_{i}=\frac{N_{0}}{2^{i}}\right)$$

(6) $$\;\;\;\;\;\;\;\;\;\;\;P_{Hi}=\;\;\overset{N_{i}-1}{\underset{k=0}{\sum }}{\left(DataH\left[k\right]\right)}^{2}\;\;\;\;\;\;\;\;\;\;\left(N_{i}=\frac{N_{0}}{2^{i}}\right)\;\;$$

(7) $$DataHS{\left[n\right]}_{i}=LPF\left[\cos\left(2\cdot \pi \cdot \frac{f_{si}}{2}\cdot n\right)\cdot DataH{\left[n\right]}_{i}\right]$$

(8) $$B_{i}=\{\begin{array}{c} 0\;\;\;\;\;\;\;\;\;\;\;\;\;\;\;\;\;\;\;\;\;\;\;\;\;\;\;\;\;\;\;\;\;\;\;\;when\;\;\;P_{Li}\geq P_{Hi}\;\;\;\\ 1\;\;\;\;\;\;\;\;\;\;\;\;\;\;\;\;\;\;\;\;\;\;\;\;\;\;\;\;\;\;\;\;\;\;\;\;\;others\;\;\;\;\;\;\;\;\;\;\;\;\;\;\;\;\;\;\;\; \end{array}\;\;$$

(9) $$P_{max}=\;\begin{array}{c} arg\\ k \end{array}MAX\left({\left\{X{\left[k\right]}_{L}\right\}}^{2}\right)$$

(10) $$\;\;\;\;\;\;\;\;\;\;\;\;\;\;\;\;\;\;f_{bias,i}=f_{bias,i-1}+B_{i}\cdot \frac{N}{2^{i+1}}\;\;\;\;\;\left(i=0,1,\ldots ,L\right)$$

(11) $$P_{est}=f_{bias,L}+P_{max}$$

(12) $$R_{est}=\frac{P_{est}\cdot \frac{f_{S}}{N}\cdot T_{b}\cdot C}{2\Delta f}$$

In Figure 3, *L* is the iteration number of loop determined by a control register, which can be adjusted depending on the number of points of FFT module and the precision and the cover range of LiDAR system. Where *x_ADC_*[*n*] is the *n*-th sampled data coming from ADC by the sampling speed of *f_S0_* and consists of *N*_0_ samples during the chirp duration; *N*_0_ is the original number of FFT points, which has to be used for FFT operation in the existing algorithm and should has a large value, such as 8192 without the proposed algorithm. While, *x*[*n*]*_i_* denotes the down sampled data in the *i*-th loop, which consists of *N_0_/2^i^* samples and is sampled by the sampling speed of *f_Si_*(*= f_S0_/*2*^i^*). By using Equations (3) and (4), *DataL*[*n*]*_i_* and *DataH*[*n*]*_i_* are the *n*-th sample data produced from the LPF and the HPF of *x*[*n*]*_i_*, respectively. The frequency band of *DataH*[*n*]*_i_* has to be shifted by *f_Si_/*2, since the signal on the higher frequency band has to be moved to lower band for the next loop operation. Prior to band selection, *DataH*[*n*]*_i_* is converted to *DataHS*[*n*]*_i_* by Equation (7) in the DDC block. The DDC block contains an image rejection filter for removing the image signal generated after the down converting processing. The power measurements (*P_Li_* and *P_Hi_*) for the filtered data, *DataL*[*n*]*_i_* and *DataH*[*n*]*_i_*, are also performed by utilizing Equations (5) and (6) for band selection, respectively.

The Data and Frequency bias value selection block compares *P_Li_* and *P_Hi_*, selects the signal with the highest power between *DataL*[*n*]*_i_* and *DataHS*[*n*]*_i_*. *DataS*[*n*]*_i_* is the selected data with higher energy between *DataL*[*n*]*_i_* and *DataHS*[*n*]*_i_* in the *i*-th loop. In order to understand which of two band signals is selected and to calculate the original bin position of FFT after the iteration process, the Data and Frequency bias value selection block generates the information of frequency band selection in the *i*-th loop, *B_i_*, which can be given in Equation (8).

*DataS*[*n*]*_i_*is down-sampled by 2:1 decimation process and is converted to *DataD*[*n*]*_i_* for the next loop operation. *DataD*[*n*]*_i_* is the same as *x*[*n*]*_i+_*_1_, which is the input data of the *i*+1-th loop operation and the number of samples is reduced to a half of the previous loop’s input data, *x*[*n*]*_i+_*_1_. If the iteration of loop is performed for *L* times, then the FFT operation is performed for the reduced *N_0_/*2*^L^ s*ample data, and the frequency-domain data, *X*[*k*]*_L_*, is generated. By using the frequency-domain data, *P_max_* is obtained from Equation (9), which is a bin number with the maximum energy value. Since *P_max_* is the value obtained from the results of FFT processing with the reduced sample data, *P_max_* has to be adjusted by using the bias values, *B_i_*, stored during the loop operation. The adjusted bin position, *P_est_*, with the maximum energy value can be calculated by using Equations (10) and (11). *P_est_* can be the same value as the estimated bin position of the FFT having the maximum energy value in the existing algorithm, which has to use the large number of FFT points. By using Equation (12), finally, the estimated distance to a target, *R_est_*, can be obtained.

## 4. ST-CFAR Method for Improved Distance Information Extraction

This section proposes an ST-CFAR algorithm that obtains distance information with better performance than the resolution of the distance determined by the Chirping Band Width in the post-processing technique of the proposed DDC FMCW LiDAR system.

To perform an FFT for beat frequency analysis, if the beat frequency extends to a decimal multiple of the FFT bin, various patterns occur at the integer multiple of the FFT bin. It is possible to extract improved distance information by grasping the characteristics of these patterns and back-estimating at what distance the FFT bin minority multiple signal is located.

**Figure 4:**
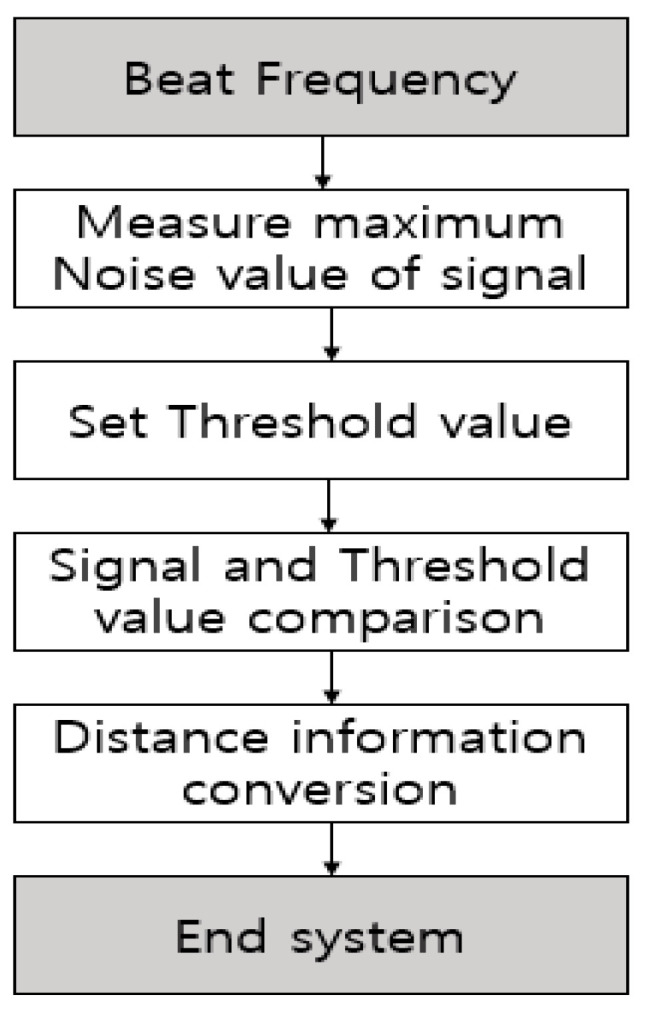
Flow Chart of ST-CFAR Algorithm.

The flowchart of the algorithm is the same as in Figure 4. After completing the restore process proposed in Section 3, set a threshold to find the maximum noise value. After that, the threshold value is set based on the user’s set value, and the pattern generated for the value higher than the threshold value when a signal corresponding to a minority multiple of the frequency resolution is received is compared with the threshold value to obtain distance information. The algorithm ends in the process of conversion.

**Figure 5:**
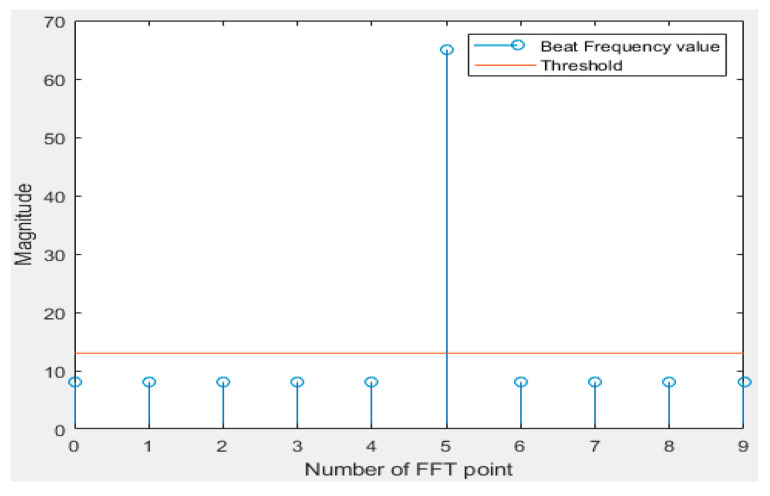
Example when the main lobe of beat frequency is located an integral multiple of the FFT bin.

**Figure 6:**
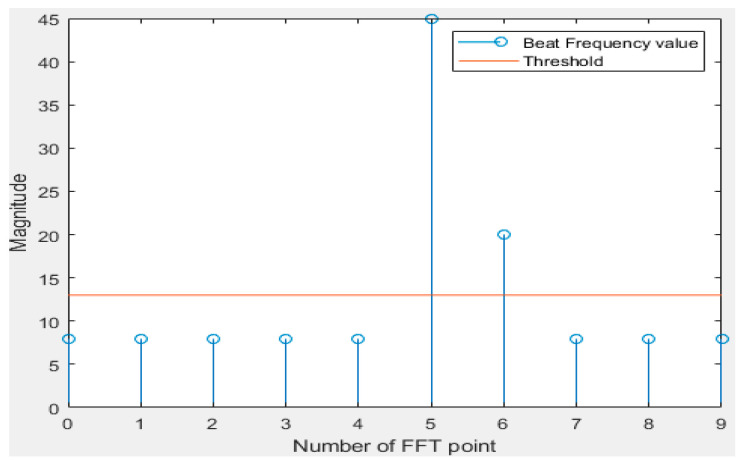
Example when the main lobe of beat frequency is located a few times the FFT bin.

Figure 5 and Figure 6 are simple examples of the frequency spectrum when the main lobe of the frequency spectrum of each received signal is located at an integral multiple and a decimal multiple of the FFT bin. Looking at Figure 5, it can be seen that only the main lobe exists because the signal corresponding to an integral multiple of the frequency resolution is input. In the case of Figure 6, since the signal came in a few times, it can be seen that it was expressed by dividing it into two FFT bins.

As an example, in the case of a system with a distance resolution of 3 cm, it can be seen at a frequency interval corresponding to 3 cm of the FFT bin interval. In the case of Figure 5 above, it can be seen that the signal corresponding to exactly 15 cm is included. Figure 6 shows that the signal corresponding to 16 cm can be detected. Using the above examples, magnitude values can be 20 and 45 at FFT bin numbers five and six, respectively, when a signal corresponding to 17 cm comes in. Since the distance resolution of the system is 3 cm, the distance is analyzed from the pattern through the same process as above for 19 cm, 20 cm, and 21 cm. By adopting this principle, more accurate detection could be possible than the precision given by the bin spacing of FFT.

The formula for acquiring the improved resolution information is given by Equation (13). Equation (13a) is for adjusting the estimated target distance when assuming that the beat frequency of target is between *P_est_* and *P_est_* + 1. *P_est_* is obtained by Equation (11). Equation (13b) is used to calculate the target distance when the beat frequency of target is between *P_est_* and *P_est_* − 1. Equation (13c) is used when the beat frequency is placed at *P_est_*. By utilizing equation (13), the improved information of target distance, *R_est,CFAR_*, can be obtained. *Tr* is a threshold value used to decide whether the neighboring frequency bin is side-lobe information caused by sub-bin positioning of beat frequency or not, which can be controlled by software.

(13) $$R_{est,CFAR}=\{\begin{array}{ccc} \frac{\frac{\left(P_{est}+\frac{1}{3}\right)}{N}\cdot f_{s}\cdot T_{b}\cdot C}{2\Delta f} & \;when\;{\left\{X\left[P_{max}+1\right]\right\}}^{2}\geq {\left\{X\left[P_{max}-1\right]\right\}}^{2}\;\;\;\;\;\;\;\;\;\&{\left\{X\left[P_{max}+1\right]\right\}}^{2}>Tr & \left(13a\right)\\ \frac{\frac{\left(P_{est}-\frac{1}{3}\right)}{N}\cdot f_{s}\cdot T_{b}\cdot C}{2\Delta f} & \;when\;{\left\{X\left[P_{max}+1\right]\right\}}^{2}<{\left\{X\left[P_{max}-1\right]\right\}}^{2}\;\;\;\;\;\;\;\;\;\&{\left\{X\left[P_{max}-1\right]\right\}}^{2}>Tr & \left(13b\right)\\ R_{est} & \;Others & \left(13c\right) \end{array}$$

## 5. Hardware Implementation

**Figure 7:**
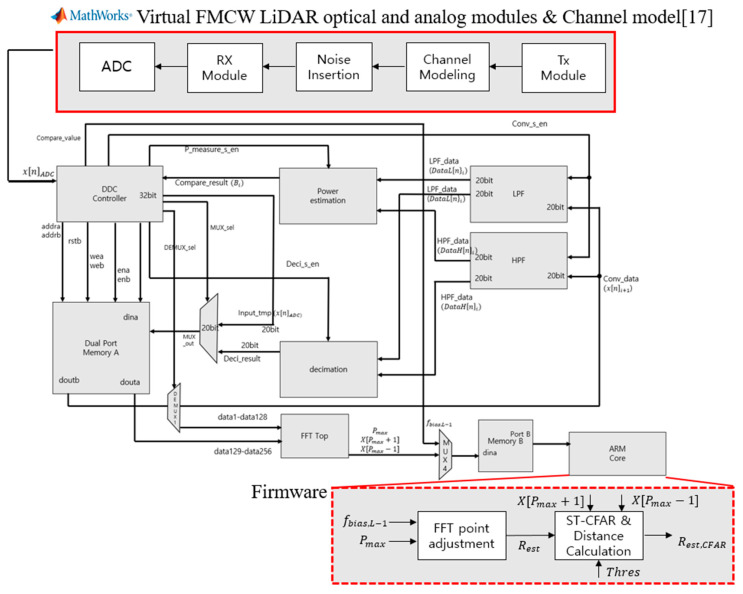
Hardware Implementation Block Diagram.

To verify this, we implemented the module using Verilog HDL, and the block diagram of the module is shown in Figure 7. For evaluation of the proposed system, virtual FMCW LiDAR module [17], channel model, noise insertion, and ADC module are designed by MATLAB Simulink. The simulation model can generate time-domain digital samples of ADC depending on the target distance, and the generated data with 8192 samples send from a PC to an ARM core on an FPGA board through wire connection. The data is observed as incoming from the ARM core to the AXI4-Lite protocol, which is stored in Memory A via the MUX. After that, the stored data are sequentially entered into the inputs of the LPF (Low Pass Filter) block, and the HPF block, respectively, and the filtered results are simultaneously entered into the power estimation block, and the Decimation block, respectively. The power estimation block sends the power comparison result to the Controller, and the Decimation block sends the LPF block result and the HPF block result to the MUX in sequence. At this time, after multiplying the HPF block result by cos data, it will be sent to MUX. The DDC controller will pass the decimated LPF result or decimated HPF result stored in Memory A to the LPF block and HPF block, based on the result output from the Power estimation block. In the same process, if 5 times are satisfied, the DDC Controller transfers the Selection signal of DEMUX 1 and sends the final 256 samples to the FFT TOP. Finally, the information to calculate the target distance, *P_max_*, *X*[*P_max_* + 1], *X*[*P_max_* − 1], and *f_bias,L_*, are fed into the ARM core, and the firmware on the ARM core can obtain the target distance value by using the received data, parameters, and Equations from (11) to (13).

**Figure 8:**
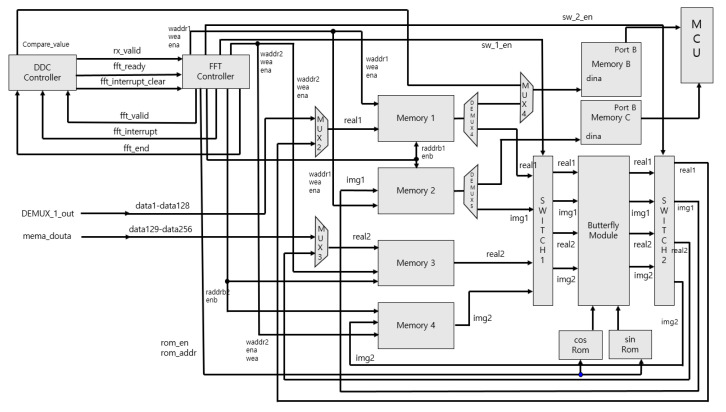
FFT Top Internal Block Diagram.

**Figure 9:**
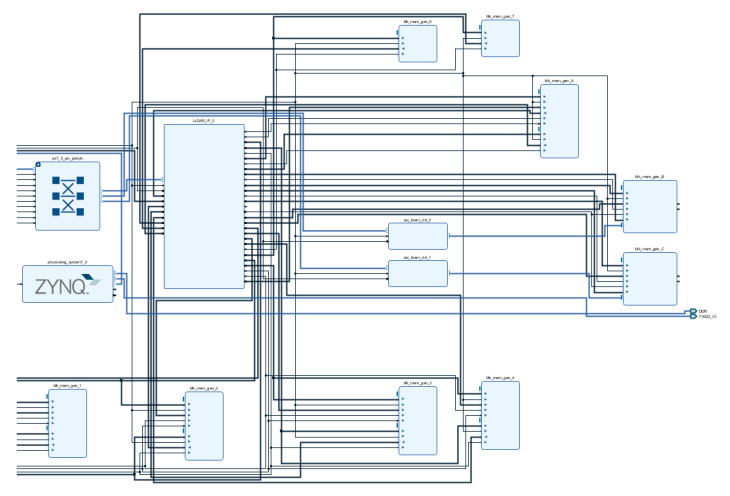
DDC FMCW LiDAR System Implemented Using VIVADO.

**Figure 10:**
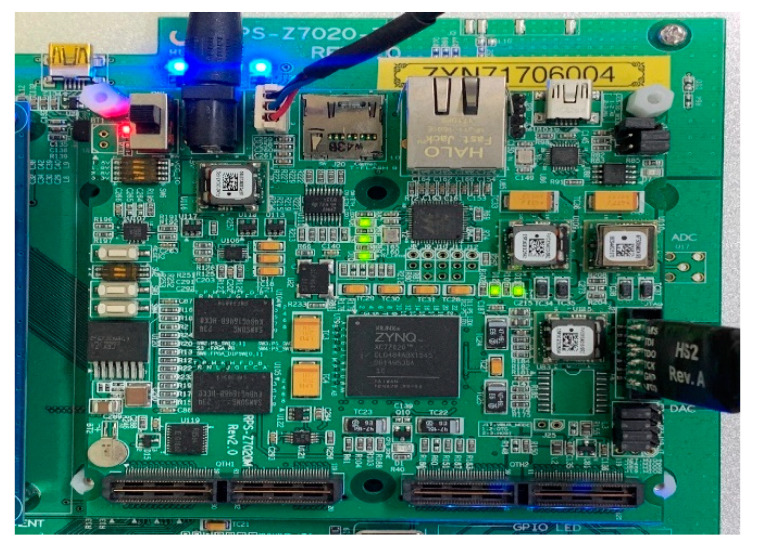
Zynq-7020 FPGA verification environment.

Figure 8 is an internal block diagram of the FFT TOP, and Figure 9 shows the implementation in VIVADO. The FFT is executed via that process, and finally Memory B stores Real data and Memory C stores Image data, which brings the data from the ARM core and sends it to the PC via UART communication. In this process, verification was performed using the Zynq-7020 board shown in Figure 10. Table 1 shows a comparison of the FPGA internal synthesis results and the number of clocks used for processing when the proposed method is applied or not. As shown in Table 1, it was found that the proposed algorithm could reduce not only the hardware complexity but the required processing time significantly.

## 6. Experiment

**Figure 11:**
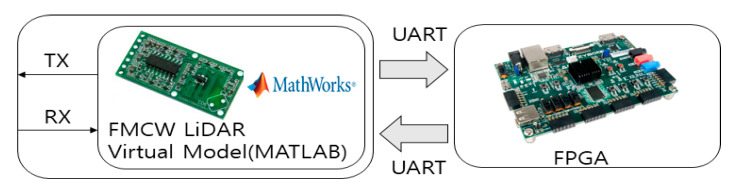
System configuration for experiments.

In this paper, in order to confirm the performance of the proposed system and algorithm, a simulation was performed using MATLAB to verify the performance. In the case of the DDC FMCW LiDAR system, we modeled it in Verilog and used FPGA to implement and verify the actual digital environment. The system configuration for the experiment is shown in Figure 11.

In this paper, the FMCW LiDAR optical module was tested with reference to existing studies [17,20]. Using MATLAB, a virtual optical module is implemented, the Beat Frequency is generated after transmission and reception, and the generated Beat Frequency data is transmitted to the FPGA via the UART standard. After that, after performing DDC and FFT processing inside the FPGA, n samples are sent to the PC via UART via the final *L*-stage. At this time, *L* is the number of loop repetitions in Figure 3, and n samples change depending on *L*.

For verifying the performance of proposed system, the target distance estimated by the proposed DDC FMCW LiDAR system is compared with the given distance from the LiDAR system to a target. At this time, the simulation environment of the system is the same as in Table 2, and the performance of this system is confirmed because the RMSE with and without application is obtained for all distances at 3 cm intervals.

(14) $$RMSE=\sqrt{\frac{1}{m}\overset{M-1}{\underset{m=0}{\sum }}{\left(R_{m}-R_{est,CFAR,m}\right)}^{2}}$$

Equation (14) is the equation for calculating RMSE, where *M* is the number of test. *R_m_* is the target distance given in the *m*-th test, and *R_est,CFAR,m_* is the target distance estimated by the proposed FMCW LiDAR system.

Table 3 shows the results of experiments using three variables in the implemented virtual model. The distance at which the maximum error occurs when the SNR of the power of the transmitted signal is 5 dB, 10 dB, and 20 dB is distinguished and the maximum error occurs when the DDC process proposed in this paper is used is calculated. In addition, the type of reflected medium was classified into Fabric and Metal, and experiments were conducted. When the medium was Fabric and only 30% of the transmission power was reflected in the home Metal, the experiment was performed assuming 90% of the transmission power. Finally, we experimented with different settings for the Number of Filter Tab used for HPF and LPF.

Table 4 shows the results of calculating the RMSE for the experimental results in which the proposed algorithm was applied in this paper and the maximum error distance was displayed in Table 3 at 0.03 m. In this experiment, assuming that the number of data passed through the first ADC is 8192, the N described in Section 3 is set to 5, and the power comparison and decimation are repeated 5 times after passing through the filter, 256 samples was extracted. After that, we completed the verification of the algorithm to obtain the RMSE from 0 cm to 50 m by estimating the peak point for the first 8192 pieces of data via Equation (2) and extracting the distance information using Equation (1).

Table 5 shows the results of applying the ST-CFAR algorithm provided based on the FFT results transferred to the PC. The distance was measured by inserting frequencies at beat frequency intervals corresponding to 1 cm based on the maximum distance of 50 m and restoring the distance. The performance was confirmed by the method of measuring the distance error RMSE, and the improvement of 1.99 cm RMSE performance was confirmed when the proposed algorithm was applied to the 3 cm RMSE ratio when the proposed algorithm was not applied.

## 7. Conclusions

In this paper, we proposed an FMCW LiDAR system that can reduce the hardware load by applying the DDC (Digital Down Convert) technique to the FMCW LiDAR system. In addition, we propose ST-CFAR (Simple Threshold Constant False Alarm Rate), which is an analysis method for the frequency spectrum pattern of the received beat frequency, and verify the improved distance by the distance resolution determined by the system’s Chirping Bandwidth by extraction simulation. For the DDC FMCW LiDAR system, based on the MATLAB simulation, implement the DDC FMCW LiDAR system in Verilog for hardware verification, use the Zynq-7020 board, perform data processing, and then in MATLAB. The system was verified by post-processing.

## Figures and Tables

**Table 1:** FPGA synthesis result before and after applying DDC.

Device Type	Zynq-7020	Zynq-7020
Algorithm	8192 FFT	DDC + 256 FFT
Slice	7420	2598
Register	11,101	2927
Max. Clock Speed	100 MHz	100 MHz
Number of Clocks	53,248 CLKs	17,123 CLKs

**Table 2:** Simulation environment in the proposed system.

Chirping Bandwidth	5 GHz
Range Resolution	3 cm
Maximum Distance	50 m
Sweep Time	37.63 µs
Number of Filtering Stage	5

**Table 3:** Error output by transmission power, medium type, and Filter Tab number.

			Ver. 1	Ver. 2	Ver. 3	Ver. 4	Ver. 5	Ver. 6	Ver. 7	Ver. 8	Ver. 9	Ver. 10
Tx SNR5dB	Fabric	LPF tap	9	15	21	27	33	39	43	49	55	85
		HPF tap	9	15	21	27	33	39	43	49	55	85
		Max err. Val.	29.55 m	0.87 m	0.45 m	0.27 m	0.21 m	0.09 m	0.09 m	0.09 m	0.03 m	0.03 m
	Metal	LPF tap	9	15	21	27	33	39	43	49	55	85
		HPF tap	9	15	21	27	33	39	43	49	55	85
		Max err. Val.	0.93 m	0.15 m	0.03 m	0.03 m	0.03 m	0.03 m	0.03 m	0.03 m	0.03 m	0.03 m
Tx SNR10dB	Fabric	LPF tap	9	15	21	27	33	39	43	49	55	85
		HPF tap	9	15	21	27	33	39	43	49	55	85
		Max err. Val.	1.41 m	0.21 m	0.09 m	0.03 m	0.03 m	0.03 m	0.03 m	0.03 m	0.03 m	0.03 m
	Metal	LPF tap	9	15	21	27	33	39	43	49	55	85
		HPF tap	9	15	21	27	33	39	43	49	55	85
		Max err. Val.	0.21 m	0.03 m	0.03 m	0.03 m	0.03 m	0.03 m	0.03 m	0.03 m	0.03 m	0.03 m
Tx SNR20dB	Fabric	LPF tap	9	15	21	27	33	39	43	49	55	85
		HPF tap	9	15	21	27	33	39	43	49	55	85
		Max err. Val.	0.03 m	0.03 m	0.03 m	0.03 m	0.03 m	0.03 m	0.03 m	0.03 m	0.03 m	0.03 m
	Metal	LPF tap	9	15	21	27	33	39	43	49	55	85
		HPF tap	9	15	21	27	33	39	43	49	55	85
		Max err. Val.	0.03 m	0.03 m	0.03 m	0.03 m	0.03 m	0.03 m	0.03 m	0.03 m	0.03 m	0.03 m

**Table 4:** DDC FMCW LiDAR system application result.

Number of Target FFT Point	256 point
Distance RMSE	0.03 m

**Table 5:** Results of applying the ST-CFAR algorithm.

	RMSE	Maximum Detect Distance
Without ST-CFAR	3 cm	50 m
With ST-CFAR	1.99 cm	50 m

## References

[1] Geiger A., Lenz P., Urtasun R. Are we ready for autonomous driving? The KITTI vision benchmark suite. Proceedings of the 2012 IEEE Conference on Computer Vision and Pattern Recognition.

[2] Levinson J., Askeland J., Becker J., Dolson J., Held D., Kammel S., Kolter J.Z., Langer D., Pink O., Pratt V. Towards fully autonomous driving: Systems and algorithms. Proceedings of the 2011 IEEE Intelligent Vehicles Symposium (IV).

[3] Chen X., Ma H., Wan J., Li B., Xia T. Multi-view 3d object detection network for autonomous driving. Proceedings of the IEEE Conference on Computer Vision and Pattern Recognition.

[4] Lim K., Islam T., Kim H., Joung J. A Sybil Attack Detection Scheme based on ADAS Sensors for Vehicular Networks. Proceedings of the 2020 IEEE 17th Annual Consumer Communications & Networking Conference (CCNC).

[5] Goodin C., Carruth D.W., Doude M., Hudson C.R. (2019). Predicting the Influence of Rain on LIDAR in ADAS. Electronics.

[6] Abdelazim S., Santoro D., Arend M., Moshary F., Ahmed S. (2018). A Hardware Implemented Autocorrelation Technique for Estimating Power Spectral Density for Processing Signals from a Doppler Wind Lidar System. Sensors.

[7] ChangalVala R., Malik H. (2019). LiDAR Data Integrity Verification for Autonomous Vehicle. IEEE Access.

[8] Yoneda K., Tehrani H., Ogawa T., Hukuyama N., Mita S. Lidar scan feature for localization with highly precise 3-D map. Proceedings of the 2014 IEEE Intelligent Vehicles Symposium Proceedings.

[9] Mirzaei F.M., Kottas D.G., I Roumeliotis S. (2012). 3D LIDAR–camera intrinsic and extrinsic calibration: Identifiability and analytical least-squares-based initialization. Int. J. Robot. Res..

[10] Lim K., Treitz P., Wulder M., St-Onge B., Flood M. (2003). LiDAR remote sensing of forest structure. Prog. Phys. Geogr. Earth Environ..

[11] Knigge A., Klehr A., Liero A., Fricke J., Maasdorf A., Zeghuzi A., Wenzel H., Trankle G. (2019). Wavelength stabilized 905 nm diode lasers in the 100 W class for automotive LiDAR. The European Conference on Lasers and Electro-Optics.

[12] Gregorio E., Rocadenbosch F., Sanz R., Rosell-Polo J.R. (2015). Eye-Safe Lidar System for Pesticide Spray Drift Measurement. Sensors.

[13] Tsang H.K., Penty R., White I., Grant R.S., Soole J.B.D., Leblanc H.P., Andreadakis N.C., Bhat R., Koza M.A., Sibbett W. (1991). Two-photon absorption and self-phase modulation in InGaAsP/InP multi-quantum-well waveguides. J. Appl. Phys..

[14] Tsang H.K., Wong C.S., Liang T.K., Day I.E., Roberts S.W., Harpin A., Drake J., Asghari M. (2002). Optical dispersion two-photon absorption and self-phase modulation in silicon waveguides at 1.5 μm wavelength. Appl. Phys. Lett..

[15] Yin L., Agrawal G.P. (2007). Impact of two-photon absorption on self-phase modulation in silicon waveguides. Opt. Lett..

[16] Lee C.C. (1972). Two-Photon Absorption and Photoconductivity in GaAs and InP. Appl. Phys. Lett..

[17] Gao S., Hui R. (2012). Frequency-modulated continuous-wave lidar using I/Q modulator for simplified heterodyne detection. Opt. Lett..

[18] Zhang F., Yi L., Qu X. (2020). Simultaneous measurements of velocity and distance via a dual-path FMCW lidar system. Opt. Commun..

[19] Okano M., Chong C. (2020). Swept Source Lidar: Simultaneous FMCW ranging and nonmechanical beam steering with a wideband swept source. Opt. Express.

[20] Dar S.A., Qazi G. (2020). Investigation and comparison of sensitivity of LiDAR to laser physical parameters at 750 m using different detection techniques. Optik.

